# Surgical Treatment of Saccular Extracranial Carotid Artery Aneurysm

**DOI:** 10.1155/carm/4459572

**Published:** 2026-04-20

**Authors:** Henrique Salles Barbosa, Renata Salles Barbosa, Aline Cristina Pavani, Renata Sydio de Souza, Marina Tambasco Freire Vicente, Mariana Fernandes Carvalho

**Affiliations:** ^1^ Department of Surgery, Medicine School, Federal University of Juiz de Fora, Juiz de Fora, Minas Gerais, Brazil, ufjf.br; ^2^ Department of Vascular Surgery, Holy House of Mercy of Juiz de Fora, Juiz de Fora, Minas Gerais, Brazil; ^3^ Medicine School, University President Antônio Carlos, Juiz de Fora, Minas Gerais, Brazil; ^4^ Department of Anesthesiology, Holy House of Mercy of Juiz de Fora, Juiz de Fora, Minas Gerais, Brazil; ^5^ General Surgery Residency, Federal University of Juiz de Fora, Juiz de Fora, Minas Gerais, Brazil, ufjf.br

**Keywords:** carotid arteries, carotid artery diseases, saccular aneurysm

## Abstract

Extracranial carotid artery aneurysms (ECAAs) are an increase of 50% or more in the diameter of the carotid artery and classified by location. They are rare and most are located in the internal carotid artery or carotid bifurcation. Diagnosis is incidental or by the presentation of a pulsatile neck mass or neurological symptoms. There are no universal recommendations regarding its treatment. Male, 72 years old, with a pulsatile cervical mass on the left. No history of previous trauma. Angioresonance and angiography identified a saccular aneurysm in the carotid bulb, measuring 1 × 0.7 cm. Referred for open surgical treatment, which was performed under general anesthesia and by longitudinal anterior cervicotomy, endoaneurysmorrhaphy, and bovine pericardium patch. There was an uneventful postoperative recovery and hospital discharge on the 3rd postoperative day. ECAAs represent between 0.4% and 1.9% of all peripheral aneurysms. They are more prevalent in men, with an average age at diagnosis of 53 ± 17 years. They can be divided into fusiform or saccular. The most common etiology of ECAA is atherosclerosis (50% of cases). ECAAs can be classified according to Attigah into Types I to V, based on the segment involved. Half of the patients with ECAAs have symptoms at diagnosis. Presentation may include a pulsatile neck mass, cervical discomfort, headache, stroke, or neurological deficits. Although rare, rupture can occur. The indication for correction of ECAAs is based on the risk of cerebral ischemia. Duplex ultrasound is the first option for diagnosis. Angiotomography, angioresonance, and angiography are options as confirmatory examinations. The techniques for repairing ECAAs are endovascular, hybrid or open: stents, embolization, or correction by arterial resection and reconstruction or patch. ECAAs are rare and have potential complications due to cerebral thromboembolism. ECAA repair is possible through open or endovascular techniques.

## 1. Introduction

Extracranial carotid artery aneurysms (ECAAs) are defined as a 50% or greater increase in carotid artery diameter and are classified based on their location [1–3]. ECAAs are extremely rare, representing less than 1% of all arterial aneurysms and under 2% of peripheral aneurysms [2–5]. The majority are found in the internal carotid artery or at the level of the carotid bifurcation [1, 4, 5]. Diagnosis may be incidental in asymptomatic patients or established during evaluation of a pulsatile neck mass or neurological symptoms [6–8].

The limited experience reported by different centers, along with heterogeneity in etiology, aneurysm morphology, and available surgical techniques, has prevented the establishment of universal treatment guidelines in the literature [1, 9, 10]. Although carotid endarterectomy is a very frequently performed procedure, carotid aneurysms are very rare and their repair pose a unique therapeutic challenge [11]. Complications are far more common, including cranial nerve injuries [12]. We hereby describe a successful surgical repair of an asymptomatic carotid aneurysm.

## 2. Case Report

Male, 72 years old, with a previous history of systemic arterial hypertension and Stage III chronic kidney disease, sought medical attention after noticing a pulsatile mass on the left side of his neck for approximately 1 month. There was no prior history of trauma, cervical surgical, or endovascular interventions, nor fine‐needle aspiration biopsy. Physical examination revealed a palpable, pulsatile mass in the left cervical region. No neurological deficits were observed.

**FIGURE 1:**
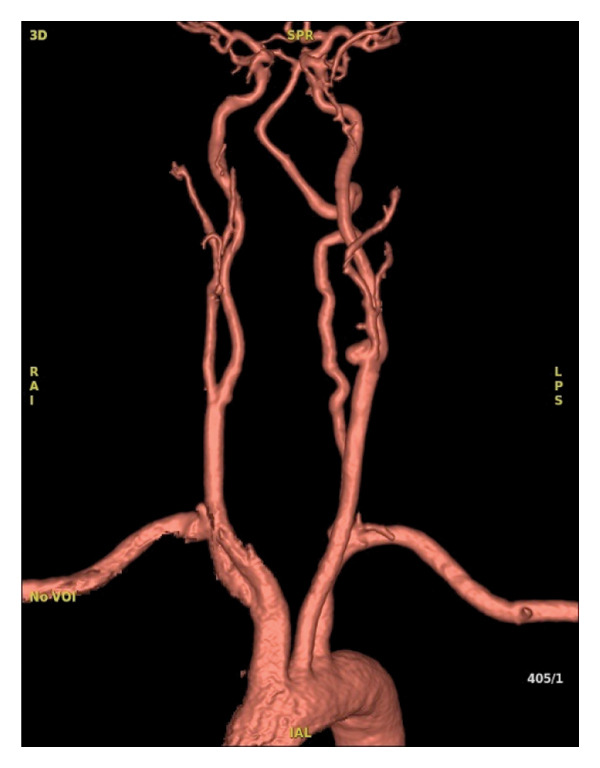
Preoperative arterial resonance angiography.

**FIGURE 2:**
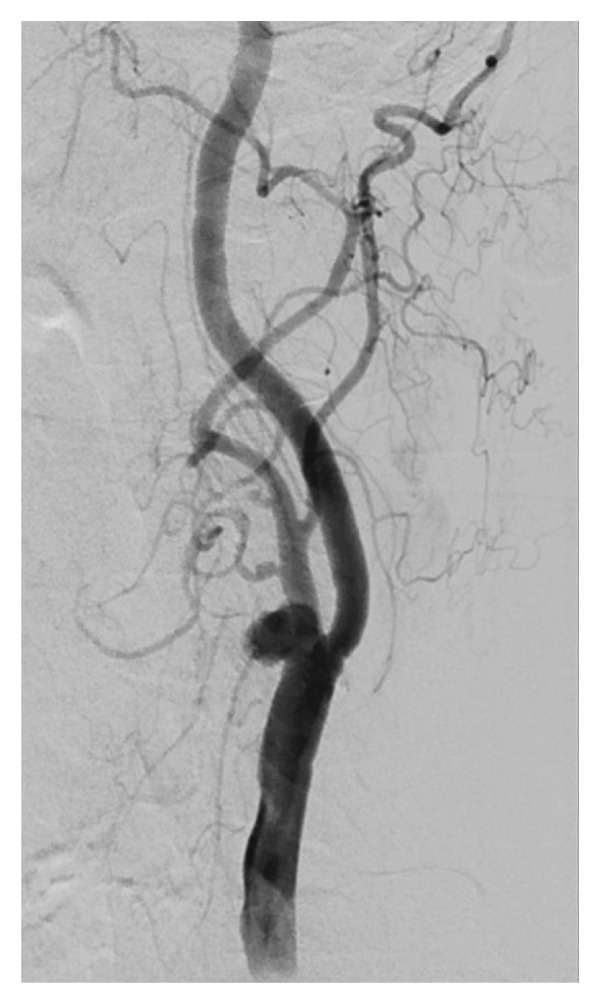
Digital subtraction angiography.

**FIGURE 3:**
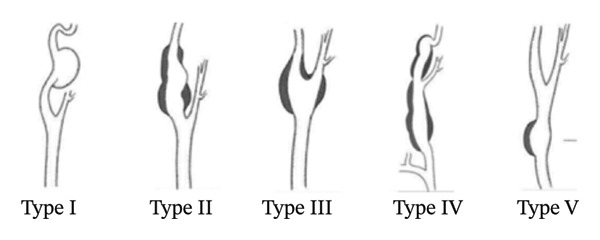
Attigah classification of carotid aneurysms. Reprinted from annals of vascular surgery. 2022; 83: 349–357, Hoffman ME, Squiers JJ, Hamandi M, Lanfear AT, Calligaro KD, Shutze WP, systematic review of the influence of anatomy and aneurysm type on treatment choice and outcomes in extracranial carotid artery aneurysms, page 351, copyright 2022, with permission from Elsevier.

As complementary investigations, magnetic resonance angiography (Figure 1) and digital subtraction angiography (Figure 2) were performed, revealing a saccular aneurysm located at the carotid bulb and the origin of the left internal carotid artery, measuring 1.0 × 0.7 cm. According to the Attigah classification (Figure 3), it was a Type III aneurysm. Due to the short and wide neck of the aneurysm, along with the involvement of the carotid bifurcation, the case was deemed unsuitable for endovascular treatment and was referred for surgical management.

**FIGURE 4:**
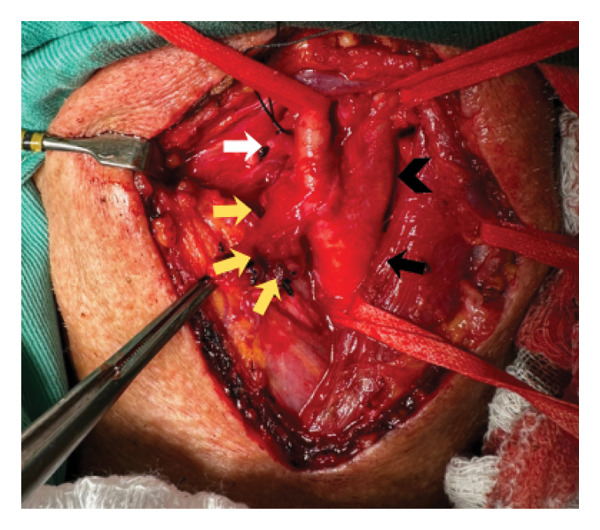
Intraoperative: dissection of left common (black arrow), internal (black arrowhead), external carotid (white arrow) arteries, and the saccular aneurysm (yellow arrows).

**FIGURE 5:**
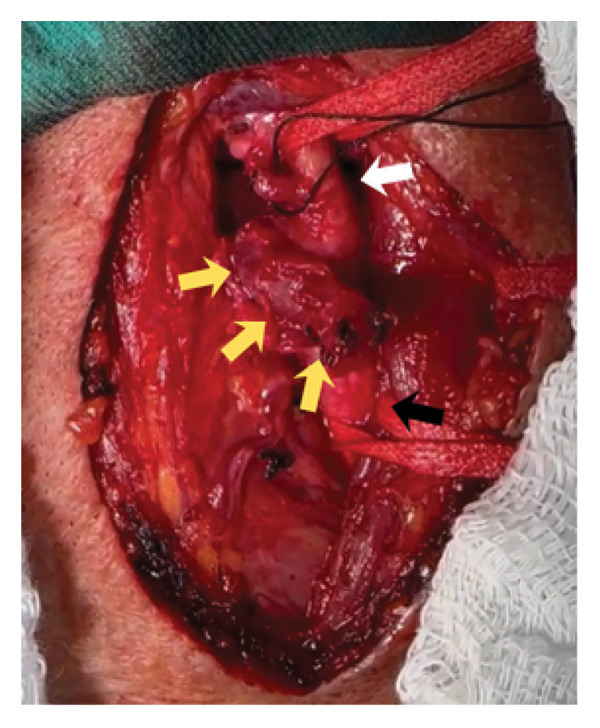
Exposure of saccular aneurysm (yellow arrows) after clockwise rotation of carotid common (black arrow) and external (white arrow) arteries.

**FIGURE 6:**
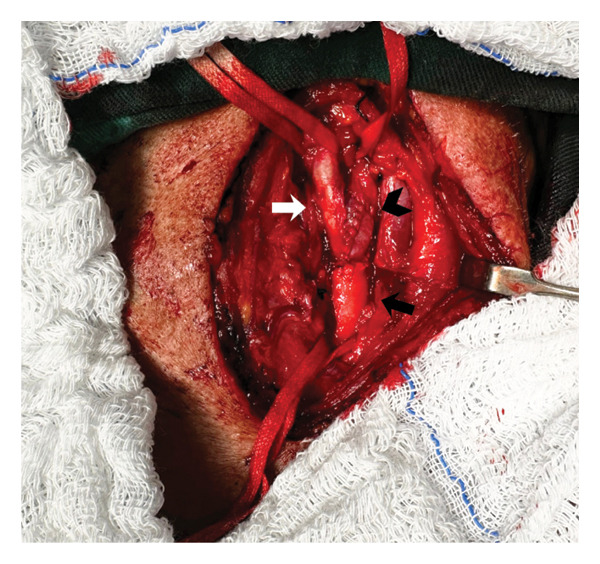
Final appearance of external (white arrow), internal (black arrowhead), and common (black arrow) carotid arteries after endoaneurysmorrhaphy and angioplasty with bovine pericardial patch.

The surgical procedure was performed under general anesthesia with nasotracheal intubation to facilitate cervical access. Left longitudinal anterior cervicotomy was performed. After vessel dissection and systemic heparinization, arterial clamping was performed, followed by a longitudinal arteriotomy of the carotid bulb and the origin of the internal carotid artery. Then, endoaneurysmorrhaphy was performed with Prolene 6‐0. Angioplasty of the carotid bulb and the origin of the internal carotid artery was performed using a bovine pericardial patch (Figures 4, 5, and 6). Finally, the vascular clamps were released in the usual manner. Intraoperative high‐frequency Doppler confirmed patency of the internal and external carotid arteries, with laminar flow and complete exclusion of the aneurysm. Closed drainage was performed with a suction drain.

**FIGURE 7:**
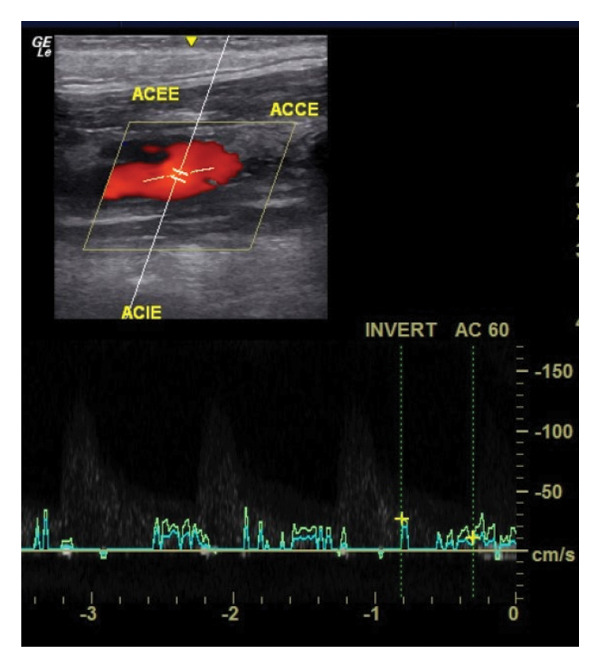
Postoperative Doppler performed at 30 days. (ACCE: left common carotid artery. ACIE: left internal carotid artery. ACEE: left external carotid artery).

Postoperative recovery was uneventful, including one day in the Intensive Care Unit and discharge on Postoperative Day 3. Postoperative Doppler monitoring at 30 days showed patency of the carotid branches, with no significant dilations or stenosis (Figure 7). Complete exclusion of the aneurysm was observed. No postoperative neurological deficits were identified. A definitive histopathological assessment of the underlying etiology was precluded by the insufficient quantity and fragmentation of the specimen.

## 3. Discussion

An artery is considered aneurysmal when it exhibits a dilation of at least 50% compared to its normal diameter [1–3, 7]. This concept also applies to ECAAs, although there is no universally accepted reference diameter that defines an aneurysmal threshold [1]. Jong et al. define an ECAA based on the following parameters: A carotid bulb aneurysm is characterized by a 50% increase in diameter relative to the normal common carotid artery or a 100% increase compared to the internal carotid artery diameter [13]. An aneurysm of the internal carotid artery is characterized by a diameter increase of more than 20% compared to the adjacent normal segment [13].

ECAAs are rare in both incidence and prevalence, accounting for 0.1%–3.7% of carotid procedures in reference centers and only 0.4%–1.9% of all peripheral aneurysms [5, 8–10, 14, 15]. They most commonly involve the internal carotid artery, followed by the common carotid artery [7]. Prevalence is higher in men (1.9:1), and the mean age at diagnosis is 53 ± 17 years [7].

They can be classified by shape as either fusiform or saccular. Fusiform aneurysms most commonly involve the carotid bifurcation and often present bilaterally. Saccular aneurysms are usually unilateral and predominantly affect the middle segment of the internal carotid artery [5].

ECAAs have several possible etiologies. More than half of the cases demonstrate the presence of atherosclerosis [2, 5, 7]. Other causes include arterial dissection, cervical trauma, prior local surgery, and exposure to local radiation [2]. Systemic or vascular conditions such as collagen‐related diseases, Takayasu arteritis, fibromuscular dysplasia, cystic medial necrosis, Behçet’s disease, idiopathic medial arteriopathy, and thrombophilias (e.g., Protein C and S deficiencies, Antithrombin III deficiency) have also been implicated [2]. Infectious etiologies, including mycotic aneurysms—most commonly caused by *Salmonella* or *Treponema pallidum (syphilis)*—are less frequent but recognized contributors [5–7, 9]. Among the clinical conditions most associated with ECAAs are hypertension, coronary artery disease, peripheral arterial disease, smoking, and dyslipidemia [1, 7].

ECAAs can be classified based on the arterial segment involved. According to Attigah (Figure 3), ECAAs are classified as follows: Type I—isolated aneurysm of the internal carotid artery, Type II—complete aneurysm of the internal carotid artery involving the bifurcation, Type III—aneurysm of the carotid bifurcation, type IV—combined aneurysm of the common and internal carotid arteries, and Type V–isolated aneurysm of the common carotid artery [1].

Approximately half of the patients with ECAAs are symptomatic at the time of diagnosis [1]. Clinical presentation is variable and may include a pulsatile cervical mass, neck discomfort, headache, stroke secondary to thromboembolism, and neurological deficits related to cranial nerve compression—such as dysphagia, hoarseness, tongue weakness, and tinnitus. Although rare, rupture can still occur [1, 7, 14–16]. Correction is indicated due to the potential risk of cerebral ischemia caused by arterial embolism or thrombosis [4, 7, 14–17].

Larger aneurysms are associated with an increased incidence of cranial nerve palsy [12, 14, 16]. Cerebral ischemia, in turn, may occur in both large and small aneurysms, typically as a result of thromboembolic events [14–16, 18]. Observational studies report an average annual increase in aneurysmal sac diameter of approximately 2 mm [16, 17].

Doppler ultrasound is considered the initial diagnostic tool of choice. Angiotomography and angioresonance remain as confirmatory tests for aneurysm, as well as for differential diagnosis [7]. Like ultrasound, these imaging modalities can identify intraluminal thrombus and delineate the aneurysm’s relationship with surrounding structures. Digital subtraction angiography, despite its invasive nature, remains the gold standard for the identification of carotid aneurysms and detailed assessment of flow characteristics and vascular anatomy.

Treatment options for ECAAs include nonoperative management, as well as open, endovascular, and hybrid surgical approaches [7]. Conservative treatment may be considered in selected asymptomatic patients and typically consists of antithrombotic therapy [19]. In this context, a review of asymptomatic patients managed conservatively reported an ipsilateral stroke rate of approximately 1.1 per 100 patient‐years [19]. However, the available evidence remains limited, as it is largely based on observational data with relatively short follow‐up periods [19].

In contrast, symptomatic aneurysms are associated with a significantly worse prognosis, with reported stroke rates reaching 50% [12]. In such cases, a conservative approach is generally considered less appropriate [10, 12, 19].

Conservative management of ECAAs may involve anticoagulant or antiplatelet therapy. Exclusive pharmacological treatment, although it may reduce the risk of cerebral ischemic events, does not resolve the aneurysm [4]. The substantial risk of thrombus formation, distal embolization, and cerebral ischemia therefore remains [4]. In patients managed conservatively, cerebral ischemia due to thromboembolism is the most frequent complication [7]. Due to the possibility of ischemic events with permanent neurological deficits, a nonoperative approach is not justified in most cases [4].

The optimal interventional procedure of ECAAs remains a matter of ongoing debate [20, 21]. Open surgical repair has traditionally been considered the gold standard, offering durable results with low recurrence rates, although it carries a risk of cranial nerve injury [12, 13, 19].

In recent years, endovascular techniques have emerged as an increasingly adopted alternative, particularly in patients with significant comorbidities, high surgical risk, or aneurysms located in anatomically challenging regions such as the distal internal carotid artery [6, 12, 20]. Endovascular options include the use of covered stent grafts to exclude the aneurysm from the circulation, as well as bare‐metal stents or flow‐diverting devices in selected cases [6, 21]. These approaches offer the advantages of reduced operative time, avoidance of extensive surgical exposure, and lower immediate perioperative morbidity.

While open surgery remains the predominant approach, the use of endovascular interventions is steadily increasing. Available evidence suggests that both techniques yield comparable outcomes and are associated with low rates of mortality and neurological complications [15–17]. However, each strategy has inherent limitations and potential complications [19].

Open repair is associated with a higher risk of cranial nerve injury, whereas endovascular treatment raises concerns regarding long‐term durability, in‐stent restenosis or thrombosis, and the need for prolonged antiplatelet therapy [6, 20]. Current evidence, largely derived from case series and systematic reviews, supports the safety and efficacy of both approaches when appropriately selected [12, 19]. Therefore, treatment decisions should be individualized, taking into account patient characteristics, aneurysm morphology, and institutional expertise, as no consensus guidelines currently define a single preferred approach [12, 19].

In the open surgical approach, the techniques used include (1) aneurysm resection with patch angioplasty and (2) resection followed by arterial reconstruction using end‐to‐end anastomosis. For endovascular procedures, the implantation of covered stents and bare‐metal stents—either alone or in combination with embolization—is among the most common techniques [14].

ECAAs classified as Types I and V according to Attigah are the most frequently managed using endovascular techniques [2, 14]. Regarding open surgery, there are reports of its successful use in treating ECAAs across all Attigah classification types [14]. Technique selection depends on the anatomical characteristics of the aneurysm, including its location, the presence of arterial kinking, and associated comorbidities [15]. Complete resection of the aneurysm followed by reconstruction is still considered the most effective strategy for definitive disease elimination [16, 17].

No standardized guidelines or consensus currently exist for the management of ECAAs. A more distal location of the aneurysm favors endovascular treatment, as well as prior ipsilateral cervical surgery or radiotherapy. Endovascular treatment may also be beneficial in cases of active incision‐site infection or when patients are not suitable candidates for open surgery [17, 20].

Surgical complications may include cervical hematoma, pseudoaneurysm formation, cranial or peripheral nerve injury, perioperative cerebral ischemia, and death [7, 14, 15]. Endovascular treatment, in turn, is associated with specific complications, including endoleak, respiratory failure and neurological deficits (both related to the aneurysm’s mass effect), arterial dissection, arterial occlusion, intracerebral hemorrhage, and embolic ischemia [15, 20]. Late carotid stenosis has also been reported, as well as stent thrombosis resulting from interruption of antithrombotic therapy [21].

ECAAs are rare and have the potential to cause complications due to cerebral thromboembolism. Treatment options include endovascular and open surgical approaches. Currently, there is insufficient evidence to demonstrate the superiority of one treatment method over another. However, open surgery is considered the standard approach for definitive treatment in clinically suitable patients with favorable anatomical conditions.

## Funding

The authors received no financial support for the research, authorship, and/or publication of this article.

## Ethics Statement

The study was conducted in accordance with the Declaration of Helsinki, and the protocol was approved by the Ethics Committee of Participant Institution.

## Consent

Written informed consent was obtained from the patient for the publication of this study and accompanying images.

## Conflicts of Interest

The authors declare no conflicts of interest.

## Data Availability

The data that support the findings of this study are available from the corresponding author upon reasonable request.
